# Microbiological and inflammatory profiles in diabetic foot infection: A severity-stratified analysis

**DOI:** 10.1097/MD.0000000000049558

**Published:** 2026-07-10

**Authors:** Ya-li Zhu, Ting Bai, Yan Chang, Shu-ting Cui, Rui Cao, Hai-mei Du

**Affiliations:** a Department of Endocrinology, Affiliated Hospital of Yan’an University, Yan’an, Shaanxi, China; b Yan’an University, Yan’an, Shaanxi, China.

**Keywords:** antibiotic sensitivity, bacterial spectrum, diabetic foot infection, inflammatory markers

## Abstract

This study analyzed the bacterial spectrum, antibiotic susceptibility profiles, and serum levels of human neutrophil lipocalin (HNL) and procalcitonin (PCT) in patients with diabetic foot infection (DFI) stratified by infection severity. A retrospective analysis was conducted on clinical data from DFI patients admitted to the Department of Endocrinology and Metabolism at Yan’an University Affiliated Hospital between January 2018 and May 2024. Based on the 2015 Infectious Diseases Society of America/ International Working Group on the Diabetic Foot classification system, patients were classified into mild, moderate, and severe infection groups. Bacterial pathogen distribution, antibiotic susceptibility, and inflammatory markers (HNL [ng/mL] and PCT [ng/mL]) were compared across groups. Among 387 screened patients, 208 (53.8%) had positive bacterial cultures. Monomicrobial infections were identified in 160 cases (77.0%), while polymicrobial infections accounted for 44 (21.2%). Gram-positive (G+) bacteria constituted 58.2% (121/208) of isolates, and Gram-negative (G−) bacteria represented 18.8% (39/208). The predominant Gram-positive (G+) bacteria were *Staphylococcus aureus* (52.1%), *Staphylococcus epidermidis* (14.9%), *Staphylococcus hominis* (5.0%), and *Staphylococcus haemolyticus* (5.0%), with *S aureus* being significantly more frequent across all severity groups (*P* < .05). Common Gram-negative (G−) isolates included *Escherichia coli* (28.1%), *Klebsiella pneumoniae* (15.4%), and *Klebsiella oxytoca* (10.3%), with no significant differences among groups. Gram-positive (G+) isolates exhibited high susceptibility to gentamicin, vancomycin, oxacillin, and linezolid, with no intergroup differences. Vancomycin susceptibility was significantly higher in moderate and severe groups compared to the mild group (*P* < .05). Gram-negative (G−) bacteria showed consistent susceptibility to ampicillin, levofloxacin, ceftazidime, cefoperazone-sulbactam, and meropenem across severity groups. Levels of HNL (ng/mL), PCT (ng/mL), white blood cell count (10^9^/L), neutrophil percentage (%), C-reactive protein (mg/L), and erythrocyte sedimentation rate (mm/h) increased significantly with infection severity (*P* < .05). However, high-sensitivity C-reactive protein (mg/L) did not differ significantly among groups. Although polymicrobial infections were increasingly observed in DFI, Gram-positive (G+) bacteria (especially *S aureus*) remain predominant. A shift toward Gram-negative (G−) pathogens was associated with higher infection severity. Empirical antibiotic treatment should be guided by local antimicrobial susceptibility patterns. The strong correlation of HNL and PCT with infection severity suggests their potential value in clinical assessment and management.

## 1. Introducton

Diabetic foot infection (DFI) is a common and serious complication of diabetes mellitus, presenting a substantial challenge to healthcare systems worldwide.^[1]^ Epidemiological studies indicate that approximately 15 to 25% of individuals with diabetes will develop a foot ulcer during their lifetime, with a significant subset progressing to infection.^[2]^ DFI represents the leading cause of nontraumatic lower limb amputations and is associated with markedly elevated mortality rates, reduced quality of life, and substantial healthcare expenditures.^[2]^ The development of DFI arises from a complex triad of peripheral neuropathy, peripheral arterial disease (PAD), and immune dysfunction, which collectively facilitate microbial colonization and impair host defense mechanisms.^[3]^

Optimal management of DFI relies heavily on the timely administration of targeted antimicrobial therapy, necessitating accurate knowledge of prevalent pathogens and their antibiotic susceptibility patterns. Current literature identifies Gram-positive (G+) bacteria, particularly *Staphylococcus aureus*, as the most frequently isolated pathogens in DFI.^[4]^ However, emerging evidence points to a shifting microbiological profile, characterized by an increasing incidence of polymicrobial infections and a growing proportion of Gram-negative (G−) bacteria in moderate to severe cases.^[5]^ This epidemiological evolution complicates empirical treatment strategies and highlights the critical need for continuous local surveillance of causative microorganisms and resistance trends.^[5]^

Accurate clinical stratification of infection severity is equally crucial for guiding therapeutic interventions and predicting outcomes. Conventional inflammatory markers, including C-reactive protein (CRP) and erythrocyte sedimentation rate (ESR), are commonly used but often lack the specificity required to reliably detect bacterial infection or accurately reflect its severity.^[6]^ This has stimulated investigation into more specific biomarkers. Human neutrophil lipocalin (HNL), a protein released from neutrophil secondary granules upon activation, has shown promise as an early indicator of acute bacterial infection.^[7,8]^ Similarly, procalcitonin (PCT), a precursor peptide hormone that increases markedly in systemic bacterial infections, aids in distinguishing bacterial from nonbacterial inflammation and in informing antibiotic stewardship.^[8]^

Despite these advances, integrated studies correlating microbiological findings, antimicrobial resistance data, and biomarker levels across well-defined DFI severity groups remain limited. Therefore, this study was designed to perform a severity-stratified analysis investigating the contemporary bacteriological etiology and antibiotic susceptibility patterns in a DFI patient cohort. A parallel objective is to quantitatively assess the dynamic changes and clinical utility of HNL and PCT as potential biomarkers for objective infection severity assessment and management guidance.

## 2. Methods

### 2.1. Study design and patient selection

This cross-sectional study retrospectively analyzed data from 208 patients diagnosed with DFI who were admitted to the Department of Endocrinology and Metabolism at Yan’an University Affiliated Hospital between January 2018 and May 2024. It was approved by the Ethics Committee of Yan’an University Affiliated Hospital (Approval No: S-S20240087). Written informed consent was obtained from all participants.

The diagnosis of DFI was established based on the 2004 diagnostic criteria issued jointly by the Infectious Diseases Society of America (IDSA) and the International Working Group on the Diabetic Foot (IWGDF).^[9]^

Patients were classified into 3 groups according to the 2015 IDSA/IWGDF infection severity grading system as follows:

No infection: absence of clinical signs or symptoms of infection.

Mild infection: infection confined to the skin and subcutaneous tissues, presenting with erythema extending 0.5 to 2.0 cm from the ulcer edge, without other causes of skin inflammation.

Moderate infection: infection involving structures deeper than the subcutaneous tissue (e.g., abscess, osteomyelitis, septic arthritis, fasciitis) or with local erythema > 2 cm from the ulcer margin, in the absence of systemic inflammatory response.

Severe infection: local infection accompanied by a systemic inflammatory response, defined as meeting 2 or more of the following criteria: body temperature > 38°C or < 36°C, heart rate > 90 beats/min, respiratory rate > 20 breaths/min, arterial partial pressure of CO_2_ < 32 mm Hg, white blood cell (WBC) count (10^9^/L) > 12.0 × 10^9^/L or < 4.0 × 10^9^/L, or presence of ≥ 10% immature neutrophils.

### 2.2. Eligibility criteria

Participants will be included in the study if they meet all of the following criteria: a confirmed diagnosis of diabetes mellitus based on standard international guidelines (e.g., those established by the American Diabetes Association or the World Health Organization), a diagnosis of DFI or ulceration according to the 2004 IWGDF/IDSA classification, and evidence of microbial infection demonstrated by a positive bacterial culture from deep tissue or purulent secretions obtained from the foot ulcer.

Individuals will be excluded from participation based on the following: presence of ulcers with etiologies other than diabetes (such as venous ulcers, arterial ulcers, malignant ulcers, or traumatic wounds) or ulcers located outside the foot region; a recent history (within 6 months) of acute cardiovascular or cerebrovascular events (including myocardial infarction or stroke), diagnosed acute hepatic failure, acute renal failure, or any other critical and uncontrolled comorbid condition that may threaten life or interfere with the study procedures.

### 2.3. Data collection and laboratory methods

Demographic and clinical data were retrieved from the Hospital Information System. Collected variables included age, sex, duration of diabetes, history of diabetic foot ulcer (DFU), diabetic peripheral neuropathy, PAD, diabetic nephropathy, and diabetic retinopathy. Laboratory parameters included bacterial culture results from ulcer secretions, antibiotic susceptibility profiles, and levels of inflammatory biomarkers such as HNL (ng/mL) and PCT (ng/mL).

### 2.4. Bacterial culture and identification

After cleansing the ulcer and removing necrotic tissue and superficial exudate with saline, deep wound secretions were collected using sterile swabs. The samples were immediately placed into sterile transport containers and delivered to the clinical microbiology laboratory for processing. Specimens were inoculated onto standardized culture media, including blood agar, MacConkey agar, and chocolate agar plates. To facilitate the growth of a wide spectrum of potential pathogens, inoculated plates were incubated at 35°C under a 5% CO_2_ atmosphere for 24 to 72 hours. Additionally, obligate anaerobic cultures were performed using appropriate anaerobic media and incubation conditions. Bacterial identification and antibiotic susceptibility testing were performed using the VITEK 2 Compact automated system (bioMérieux) in accordance with the manufacturer’s protocols.

### 2.5. Statistical analysis

Statistical analyses were conducted using SPSS version 26.0. Continuous variables were compared using Student *t*-test or nonparametric tests as appropriate, while categorical variables were analyzed using the chi-square (*χ*^2^) test. A *P* value < .05 was considered statistically significant.

## 3. Results

### 3.1. Patient selection

**Figure 1.**
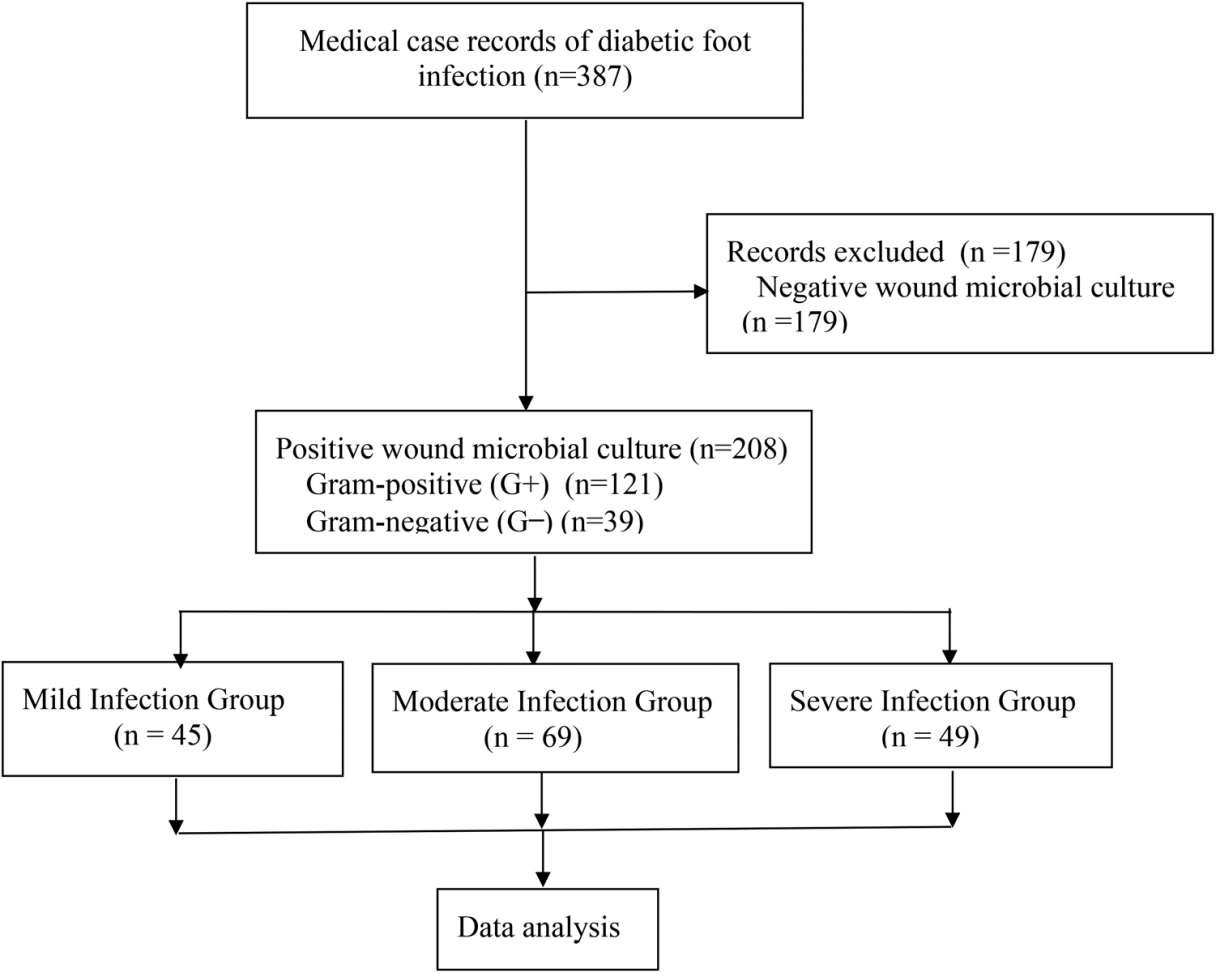
Flowchart of patient selection.

A total of 387 medical records of patients with DFI were initially screened. We excluded 179 cases with negative wound microbial cultures, resulting in 208 patients with confirmed positive bacterial cultures comprising the final analytical cohort. These culture-positive cases were stratified according to the IDSA/IWGDF classification system into 3 severity-based groups: mild (n = 45), moderate (n = 69), and severe infection (n = 94). Microbiological analysis showed that monomicrobial Gram-positive infections accounted for 121 cases (58.2%), while monomicrobial Gram-negative infections were identified in 39 cases (18.8%). The flowchart outlines the participant selection sequence and distribution across severity strata for subsequent analysis (Fig. 1).

### 3.2. Baseline demographic and clinical characteristics

A total of 208 patients were included in the analysis. The cohort comprised 163 males (78.3%) and 45 females (21.6%). Based on the severity of DFI, patients were classified into 3 groups: mild (n = 45, 21.6%), moderate (n = 69, 31.3%), and severe (n = 94, 45.1%). No significant differences were observed among the groups regarding sex, age, duration of diabetes, glycated hemoglobin levels, history of smoking or alcohol use, hypertension, cerebral infarction, PAD, diabetic peripheral neuropathy, diabetic nephropathy, or diabetic retinopathy. However, the moderate infection group exhibited a significantly higher prevalence of previous DFU and coronary heart disease compared to the mild and severe infection groups (*P* < .05). Detailed data are presented in Table 1.

**Table 1:** Comparison of patient characteristics with different degrees of infection (n, %).

Indicators	Values	Mild infection group (n = 45)	Infection grade	Severe infection group(n = 94)	*P* value
			Moderate infection group (n = 69)		
Gender (Female)	45 (21.6)	14 (31.1)	12 (17.4)	19 (20.2)	.199
Age (yrs)	63.55 ± 11.62	61.84 ± 11.32	67.29 ± 11.26	61.63 ± 11.47	.004
Duration of diabetes (yrs)	14.17 ± 6.42	14.07 ± 6.18	14.71 ± 7.85	13.83 ± 5.33	.690
Duration of DFU (mo)	2.83 ± 7.22	1.78 ± 7.27	3.23 ± 4.46	3.04 ± 8.70	.540
Smoking history	56 (26.9)	11 (24.4)	19 (27.5)	26 (27.7)	.914
Alcohol history	36 (17.3)	7 (15.6)	13 (18.8)	16 (17.0)	.898
Glycated hemoglobin	10.04 ± 2.56	9.33 ± 2.23	10.06 ± 2.31	10.37 ± 2.83	.083
Hypertension	105 (50.5)	25 (55.6)	34 (49.3)	46 (48.9)	.743
Cerebral infarction	33 (15.9)	4 (8.9)	14 (20.3)	15 (16.0)	.265
Coronary heart disease	103 (49.5)	17 (37.8)	49 (71.0)	37 (39.4)	.000
Diabetic peripheral vascular disease	171 (82.2)	34 (75.6)	57 (82.6)	80 (85.1)	.385
DPN	191 (91.8)	41 (91.1)	63 (91.3)	87 (92.6)	.941
DN	133 (63.9)	25 (55.6)	50 (72.5)	58 (61.7)	.153
DR	86 (41.3)	22 (48.9)	28 (40.6)	36 (38.3)	.489

Data are presented as mean (standard deviation) or number (%).

DFU = diabetic foot ulcer, DN = diabetic nephropathy, DPN = diabetic peripheral neuropathy, DR = diabetic retinopathy, n = number of patients.

### 3.3. Pathogen distribution and antimicrobial susceptibility profiles

#### 3.3.1. Pathogen distribution

Among the 208 patients with DFI, monomicrobial Gram-positive (G+) infections were identified in 121 cases (58.2%), monomicrobial Gram-negative (G−) infections in 39 (18.8%), and polymicrobial bacterial infections in 44 (21.2%). The predominant Gram-positive (G+) pathogen was *S aureus* (52.1%), followed by *Staphylococcus epidermidis* (14.9%), *Staphylococcus hominis* (5.0%), and *Staphylococcus haemolyticus* (5.0%). *S aureus* was significantly more prevalent across all infection severity groups (*P* < .05). Among Gram-negative (G−) isolates, *Escherichia coli* (28.1%), *Klebsiella pneumoniae* (15.4%), and *Klebsiella oxytoca* (10.3%) were most frequently identified, with no significant differences in distribution among the 3 groups (Table 2).

**Table 2:** Distribution of etiological characteristics among 3 groups.

Indicators	Patients (n,%)	Mild infection group (n = 45)	Infection Grade	Severe infection group (n = 94)	*P* value
			Moderate infection group (n = 69)		
Gram-positive (G+)	121 (58.2)	33 (73.3)	45 (65.2)	43 (45.7)	.006
Gram-negative (G−)	39 (18.8)	5 (11.1)	6 (8.7)	28 (29.8)	
Mixed Infection	44 (21.2)	6 (13.3)	17 (24.6)	21 (22.3)	
Regular Gram-positive (G+) Bacteria					
*Staphylococcus aureus*	75 (65.8)	25 (83.3)	23 (52.3)	27 (67.5)	.013
*Staphylococcus epidermidis*	18 (14.9)	0 (0.0)	16 (36.4)	10 (25.0)	
*Staphylococcus hominis*	7 (6.1)	2 (6.7)	4 (9.1)	1 (2.5)	
* Staphylococcus haemolyticus*	6 (5.3)	3 (10.0)	1 (2.3)	2 (5.0)	
Regular Gram-negative (G−) Bacteria					
*Escherichia coli*	14 (58.3)	2 (100.0)	1 (33.3)	11 (57.9)	.671
*Klebsiella pneumoniae*	6 (25.0)	0 (0.0)	1 (33.3)	5 (26.3)	
*Klebsiella oxytoca*	4 (16.7)	0 (0.0)	1 (33.3)	3 (15.8)	

Data are presented as number (%).

n = number of patients.

#### 3.3.2. Antibiotic susceptibility

Gram-positive (G+) bacteria exhibited high susceptibility rates to gentamicin, vancomycin, oxacillin, and linezolid. Although no significant differences in overall susceptibility profiles were detected among the 3 groups, sensitivity to vancomycin was notably higher in the moderate and severe infection groups compared to the mild infection group (*P* < .05). Gram-negative (G−) bacteria were most susceptible to ampicillin, levofloxacin, ceftazidime, cefoperazone-sulbactam, and meropenem, with no statistically significant differences in susceptibility patterns across the infection severity groups. Refer to Tables 3 and 4 for comprehensive results.

**Table 3:** Antibiotic susceptibility of Gram-positive bacteria (n,%).

Antimicrobial drugs	Mild infection group (n = 33)	Infection Grade	Severe infection group (n = 43)	*P* value
		Moderate infection group (n = 45)		
Gentamicin	24 (72.7)	28 (62.2)	22 (51.2)	.158
Levofloxacin	23 (69.7)	28 (62.2)	29 (67.4)	.768
Tigecycline	24 (72.7)	32 (71.1)	26 (60.5)	.438
Vancomycin	29 (87.9)	44 (97.8)	35 (81.4)	.044
Moxifloxacin	18 (54.5)	20 (44.4)	19 (44.2)	.604
Oxacillin	14 (42.4)	14 (31.1)	16 (37.2)	.585
Linezolid	28 (84.8)	39 (86.7)	35 (81.4)	.790

Data are presented as number (%).

n = number of patients.

**Table 4:** Antibiotic susceptibility of Gram-negative bacteria (n, %).

Antimicrobial Drugs	Mild infection group (n = 5)	Infection Grade	Severe infection group (n = 28)	*P* value
		Moderate infection group (n = 6)		
Gentamicin	1 (20.0)	3 (50.0)	13 (46.4)	.516
Tobramycin	1 (20.0)	2 (33.3)	10 (35.7)	.790
Ampicillin	1 (20.0)	0 (0.0)	5 (17.9)	.521
Levofloxacin	1 (20.0)	2 (33.3)	11 (39.3)	.703
Ceftazidime	3 (60.0)	5 (83.3)	14 (50.0)	.323
Cefepime	4 (80.0)	5 (83.3)	15 (53.6)	.262
Cefoperazone Sodium/Sulbactam	3 (60.0)	3 (50.0)	14 (50.0)	.916
Piperacillin/Tazobactam	4 (80.0)	3 (50.0)	14 (50.0)	.454
Meropenem	4 (80.0)	5 (83.3)	22 (78.6)	.966
Imipenem (Thienamycin, also known as Tienam)	4 (80.0)	6 (100.0)	19 (67.9)	.250

Data are presented as number (%).

n = number of patients.

### 3.4. Inflammatory markers across infection severity groups

Levels of inflammatory biomarkers, including HNL (ng/mL), PCT (ng/mL), WBC count (10^9^/L), neutrophil count (NEU) (%), CRP (mg/L), and ESR (mm/h), demonstrated a significant stepwise increase with escalating infection severity (all *P* < .05; Table 5). In contrast, high-sensitivity CRP (hs-CRP) (mg/L)levels did not differ significantly among the groups.

**Table 5:** Comparison of inflammatory markers in diabetic foot patients by infection severity.

Inflammatory markers		Infection grade	Severe (n = 94)	*P* value
	Mild (n = 45)	Moderate (n = 69)		
WBC (10^9^/L)	7.25 ± 2.01	7.26 ± 1.97	9.64 ± 5.18	< .001
NEU (%)	67.02 ± 12.25	69.37 ± 9.14	74.23 ± 12.04	.001
CRP (mg/L)	27.85 ± 56.47	26.22 ± 32.80	52.85 ± 68.94	.005
hs-CRP (mg/L)	4.19 ± 4.07	5.74 ± 4.03	6.08 ± 4.20	.039
ESR (mm/h)	32.09 ± 23.67	52.64 ± 44.28	63.34 ± 43.40	< .001
PCT (ng/mL)	0.16 ± 0.30	0.19 ± 0.46	0.63 ± 2.02	.073
HNL (ng/mL)	229.75 ± 132.27	454.31 ± 182.28	390.27 ± 205.33	< .001

Data are presented as mean (standard deviation).

CRP = C-reactive protein, ESR = erythrocyte sedimentation rate, HNL = human neutrophil lipocalin, hs-CRP = high-sensitivity CRP, n = number of patients, NEU = neutrophil count, PCT = procalcitonin, WBC = white blood cell count.

Multivariate logistic regression analysis was performed using infection severity (mild, moderate, severe) as the ordinal outcome variable. Independent variables included WBC count (10^9^/L), NEU (%), hs-CRP(mg/L), ESR (mm/h), duration of diabetes, duration of diabetic foot disease, and glycated hemoglobin. The analysis revealed that elevated NEU (%) (odds ratio = 1.01, 95% confidence interval: 0.94–1.10) and ESR (mm/h) (odds ratio = 1.02, 95% confidence interval: 0.99–1.04) were associated with an increased risk of severe infection relative to mild infection. However, the risk of severe infection was not significantly associated with diabetes duration, diabetic foot duration, glycated hemoglobin, WBC (10^9^/L), NEU (%), hs-CRP (mg/L), or ESR (mm/h) in the final model. Complete regression results are provided in Table 6.

**Table 6:** Logistic regression for factors influencing infection severity in diabetic foot patients.

Indicators	*β*	SE	*P* value	OR	(95% CI)	
Moderate infection vs mild infection						
Age	0.01	0.02	.57	1.01	0.97	1.06
Coronary heart disease	1.25	0.49	.01	3.48	1.34	9.08
WBC	−0.11	0.11	.30	0.90	0.73	1.10
NEU%	0.02	0.03	.43	1.02	0.97	1.08
CRP	−0.01	0.01	.11	0.99	0.98	1.08
hs-CRP	0.02	0.07	.80	1.02	0.88	1.18
ESR	0.02	0.01	.05	1.02	1.00	1.03
HNL	0.01	0.01	.00	1.01	1.01	1.01
Severe infection vs mild infection						
Age	−0.01	0.02	.60	0.99	0.95	1.03
Coronary heart disease	0.07	0.44	.88	1.07	0.45	2.54
WBC	0.11	0.08	.19	1.12	0.95	1.32
NEU%	0.01	0.02	.60	1.01	0.97	1.06
CRP	−0.01	0.01	.63	1.00	0.99	1.01
hs-CRP	−0.07	0.07	.32	0.93	0.81	1.07
ESR	0.02	0.01	.01	1.02	1.01	1.04
HNL	0.01	0.01	.00	1.01	1.00	1.01

CI = confidence interval, CRP = C-reactive protein, ESR = erythrocyte sedimentation rate, HNL = human neutrophil lipocalin, hs-CRP = high-sensitivity CRP, NEU = neutrophil count, OR = odds ratio, SE = standard error, WBC = white blood cell count.

## 4. Discussion

DFUs represent a major cause of disability among individuals with diabetes and are strongly associated with increased mortality and overall disease burden.^[10]^ As the leading cause of nontraumatic lower extremity amputations in this population, DFUs significantly contribute to elevated morbidity and mortality rates.^[10]^ The lifetime incidence of DFUs ranges from 19 to 34%, with an annual incidence of approximately 2%.^[2]^ Globally, over 1 million diabetes-related amputations are performed each year, equivalent to 1 amputation every 20 seconds.^[11]^ The presence of infection in DFU increases the risk of lower limb amputation by 50% compared to noninfected ulcers,^[12]^ underscoring the critical importance of appropriate antibiotic use in infection control and wound healing. DFIs are common complications, and osteomyelitis (a serious bone infection) affects 50 to 60% of hospitalized patients and 10 to 20% of outpatients with DFUs.^[13]^

Previous studies indicate that Gram-positive (G+) bacteria account for approximately 70% of isolates from DFUs, with *Staphylococcus* species comprising over 35% of these.^[14]^ Consistent with reports from other centers,^[15,16]^ our findings identified *S aureus* as the most prevalent Gram-positive (G+) pathogen and *E coli* as the predominant Gram-negative (G−) organism. The distribution of pathogens varied with infection severity: in the mild infection group, Gram-positive (G+) and Gram-negative (G−) infections accounted for 73.3% and 11.1%, respectively; in the moderate group, 65.2% and 8.7%; and in the severe group, 45.7% and 29.8%. This shift toward a higher proportion of Gram-negative (G−) infections in severe cases suggests that infection severity may influence microbiological etiology. Variations in predominant pathogens have been reported across studies; for instance, Zou et al^[17]^ reported *Acinetobacter* as the most common Gram-negative (G−) genus, whereas Palomo et al^[18]^ identified *Pseudomonas aeruginosa* as the leading Gram-negative (G−) organism in hospitalized DFI patients. Differences in hygiene practices, delays in diagnosis and treatment, empirical antibiotic use, and climatic conditions may contribute to geographical variations in bacterial spectra.^[19]^

Antibiotic susceptibility analysis in our cohort indicated that Gram-positive (G+) isolates were highly sensitive to gentamicin, levofloxacin, tigecycline, vancomycin, and moxifloxacin. Notably, vancomycin sensitivity was significantly higher in moderate and severe infection groups compared to the mild group. Gram-negative (G−) bacteria demonstrated heightened sensitivity to ceftazidime, cefepime, ceftriaxone-sulbactam, piperacillin-tazobactam, and meropenem. These findings are generally consistent with those of Chang et al,^[20]^ who reported that *S aureus* isolates from DFUs were highly sensitive to rifampin, vancomycin, chloramphenicol, oxacillin, and gentamicin, while enterococci were sensitive to penicillin, vancomycin, ampicillin, and linezolid. Among Gram-negative (G−) bacteria, *E coli* showed sensitivity to imipenem, amikacin, and cefepime, and *P aeruginosa* to macrolides, beta-lactams, and quinolones. Dorr et al^[21]^ observed resistance to penicillin-beta-lactamase inhibitors among Gram-positive (G+) isolates and to piperacillin-tazobactam or carbapenems among Gram-negative (G−) organisms in DFI cases. Variations in resistance patterns across studies may reflect regional and methodological differences. However, a recent systematic review concluded that there is insufficient evidence to confirm that multidrug-resistant organisms impede DFU healing.^[22]^ Unfortunately, the limited sample size and incomplete data in our study precluded a robust statistical analysis of antibiotic resistance, highlighting the need for larger prospective studies.

Regarding inflammatory markers, our results demonstrated that levels of HNL, PCT, WBC count, NEU, CRP, and ESR increased significantly with infection severity (*P* < .05). These findings align with those of Park et al,^[23]^ who reported positive correlations between PCT/CRP levels and DFI severity. Notably, the hs-CRP assay did not demonstrate a significant gradient across severity groups, in contrast to the standard CRP test. This discrepancy may be attributed to the distinct design purposes and measurement ranges of the 2 assays. The hs-CRP test is optimized for detecting very low-grade, chronic inflammation (e.g., for cardiovascular risk stratification), and its dynamic range is often exceeded in the context of overt, acute infections like DFI, thereby diminishing its utility for severity discrimination. Consequently, the standard CRP assay, designed to quantify a broader range of higher concentrations, remains a more robust marker for monitoring the acute-phase response in DFI. HNL, stored in secondary granules of neutrophils and released upon infection, serves as a promising biomarker for acute bacterial infections.^[24,25]^ Guan et al^[26]^ further reported that HNL exhibited superior diagnostic performance for bacterial infections compared to PCT, and that combined detection of HNL, serum amyloid A, PCT, and CRP could improve discrimination between bacterial and viral etiologies. Although our study incorporated multiple inflammatory markers (including PCT, HNL, CRP, high-sensitivity CRP, and ESR) to enhance early detection and severity assessment of DFI, we did not evaluate the sensitivity, specificity, or pathogen-specific diagnostic value of HNL or PCT. Thus, no conclusions can be drawn regarding their differential diagnostic utility. Moreover, our findings demonstrate a quantitative association between serum HNL and PCT levels and DFI severity; however, this study was not powered to establish definitive clinical cutoff values. The derivation and validation of specific biomarker thresholds to objectively discriminate between mild, moderate, and severe infection grades remain a critical objective for future investigations. Such validated cutoffs could significantly enhance clinical decision-making by providing an objective rationale for guiding antibiotic initiation, intensification, or de-escalation. Therefore, well-designed prospective studies with larger cohorts are needed to derive and validate these thresholds, which would facilitate the translation of these biomarkers into practical tools for the stratified management of DFI.

This study has several limitations. First, as a retrospective single-center study limited to inpatients, the research may not capture variations in outpatient populations or across institutions with different demographic and clinical profiles, which restricts its external validity. Second, the lack of systematic data on prior antibiotic exposure and hospitalization history may have affected the observed pathogen distribution and resistance patterns, leading to potential bias in the microbiological results. Third, although HNL and PCT were evaluated as potential biomarkers, the study did not assess their diagnostic accuracy (such as sensitivity, specificity, or predictive values) for differentiating pathogen subtypes, which limits the strength of conclusions about their clinical utility. Fourth, the relatively small sample size limited detailed subgroup analyses, including comparisons of outcomes between monomicrobial and polymicrobial infections or stratification by comorbid conditions. Finally, the cross-sectional design prevented longitudinal assessment of biomarker changes and their predictive value for treatment response or clinical outcomes, highlighting the need for prospective multicenter studies with standardized methods.

## 5. Conclusion

In summary, while the generalizability of this study is constrained by its limited sample size and potential analytical biases, the findings offer clinically relevant insights into the microbiological profile of DFI and support evidence-based antibiotic selection. To optimize therapeutic outcomes, it is recommended that repeated microbial culture and antibiotic susceptibility testing be performed throughout the course of DFI management to guide antimicrobial therapy. It should be noted, however, that these results are derived from a single-center inpatient cohort, which may restrict their direct applicability to outpatient settings or populations with differing demographic and clinical characteristics. Furthermore, the absence of data on prior antibiotic exposure and history of hospitalization represents a potential source of bias, highlighting the need for more comprehensive and multicenter studies in the future.

## Author contributions

**Conceptualization:** Ya-li Zhu, Ting Bai, Hai-mei Du.

**Data curation:** Ya-li Zhu, Ting Bai, Yan Chang, Shu-ting Cui, Hai-mei Du.

**Formal analysis:** Ya-li Zhu.

**Investigation:** Hai-mei Du.

**Methodology:** Ya-li Zhu.

**Project administration:** Hai-mei Du.

**Resources:** Ya-li Zhu, Ting Bai, Yan Chang, Rui Cao.

**Supervision:** Hai-mei Du.

**Validation:** Ting Bai, Yan Chang, Shu-ting Cui, Rui Cao, Hai-mei Du.

**Visualization:** Ting Bai, Yan Chang, Shu-ting Cui, Rui Cao, Hai-mei Du.

**Writing** – **original draft:** Ya-li Zhu, Ting Bai, Yan Chang, Shu-ting Cui, Rui Cao, Hai-mei Du.

**Writing** – **review & editing:** Ya-li Zhu, Ting Bai, Yan Chang, Shu-ting Cui, Rui Cao, Hai-mei Du.
